# A Qualitative Study on Young Women’s Lives Prior to and Four Years after Youth Detention: Examining the Good Lives Model’s Aetiological Assumptions

**DOI:** 10.3390/ijerph182211830

**Published:** 2021-11-11

**Authors:** Lore Van Damme, Clare-Ann Fortune, Stijn Vandevelde, Wouter Vanderplasschen, Olivier F. Colins

**Affiliations:** 1 All-Male Youth Detention Center, De Zande, 8755 Ruiselede, Belgium; lml.vandamme@gmail.com; 2 School of Psychology, Victoria University of Wellington, Wellington 6012, New Zealand; clare-ann.fortune@vuw.ac.nz; 3 Department of Special Needs Education, Ghent University, 9000 Gent, Belgium; Stijn.Vandevelde@ugent.be (S.V.); wouter.vanderplasschen@ugent.be (W.V.); 4 Department of Behavioral, Social and Legal Sciences, Örebro University, 701 82 Örebro, Sweden

**Keywords:** aetiology, desistance, follow-up studies, good lives model, women, youth detention

## Abstract

Detained female adolescents constitute a vulnerable, challenging, and understudied minority. Interventions for DFA are still dominated by risk management approaches with less focus on strength-based approaches such as the Good Lives Model (GLM). This study explored the functionality of DFA’s behaviour prior to and four years after release from detention, using the GLM as the guiding theoretical framework. A theory-driven thematic analysis was conducted of 30 in-depth interviews with former DFA (*M*_age_ = 20.80), exploring the fulfilment of their basic human needs (e.g., relatedness, independence) before and after detention. Before detention, the young women experienced multiple problems trying to fulfil multiple human needs, often contributing to poor balance in their lives and their antisocial behaviour. Although external and internal obstacles to fulfilling human needs were still present at follow-up, important improvements were noted, e.g., in the scope of their human needs and the resources available to fulfil their needs. The findings provide additional insights into the issues experienced by young women in detention and indicate there are opportunities to assist these young women, through the development of appropriate resources and capacities which provide them with appropriate means for fulfilling their needs and moving towards a personally meaningful and prosocial life.

## 1. Introduction

Detained female adolescents (DFA) are a particularly vulnerable and challenging, yet understudied, group of young people. They often grow up under adverse living conditions and often display high levels of antisocial behaviour and co-morbid psychiatric disorders, which are likely to persist into young adulthood [1,2,3,4,5]. DFA experience significantly higher rates of trauma exposure than their male counterparts [6,7], and tend to have experienced more frequent and multiple types of maltreatment [8]. In addition, they display significantly higher rates of psychopathology [4,9,10,11], more often suffering from co-morbid internalising (e.g., major depressive disorder) and externalising disorders (e.g., conduct disorder) [7]. Furthermore, previous empirical studies on DFA have indicated that female (compared to male) adolescents are more often detained for child protection reasons, and not merely because they have committed (severe) offenses [3,12,13]. When detained due to their antisocial behaviour, DFA are often detained due to their involvement in a broader range of antisocial activities, including running away from home, truancy, defiant/relentless/uncontrollable behaviour, and risky sexual behaviour than their male counterparts (e.g., prostitution) [3,12,13,14]. There is a growing consensus among clinicians and researchers about the need to develop and deliver effective treatment for this particular population [10,15]. However, rehabilitation frameworks currently guiding the treatment of DFA come with possibly important aetiological limitations, because they tend to fail to answer the crucial question of the exact aim of the behaviour (i.e., the level of functionality; “What is the person trying to achieve [with his/her behavior]?”) [16]. As such, the function or purpose of DFA’s behaviour is not well understood.

### 1.1. Aetiological Limitations: Overlooking Functionality

In the past 30 to 40 years there has been increasing interest in identifying what works in rehabilitation for youth and adults involved in the justice system [17,18,19]. Adherence to the three core principles of the Risk-Need-Responsivity (RNR) model [20,21] has been consistently found to be associated with positive treatment outcomes. Rehabilitation frameworks, such as the RNR, are however still preoccupied by a problem-oriented risk management approach, including those guiding the treatment of DFA [22,23]. Without doubt, the RNR model’s focus on dynamic risk factors for future crime, is very useful in predicting recidivism. However, these dynamic predictors of recidivism are not conceptualised as causal factors [24], which ultimately limits the RNR model’s ability to explain why people (re)offend (the etiological level) [25]. Put differently, the RNR model does not offer a thorough insight into underlying causal mechanisms or the functionality of DFA’s (re)offending [16]. In fact, one particular type of crime is likely to have a broad range of underlying functions. For example, violent offending could either reflect a search for inner peace (i.e., trying to reduce stress) or a search for independence (i.e., trying to gain status), and each requires a different set of interventions.

A similar concern applies to the available evidence in female pathways literature. This body of literature focuses on the role of traditional (e.g., poor upbringing) and gendered (e.g., trauma exposure and relational issues) risk and protective factors in explaining the development of female (re)offending over time [26,27,28]. These studies do include references to underlying functions of females’ (re)offending, however, without using a holistic, theoretical framework that explicitly starts from a functional perspective. For example, looking at Daly’s [29] street women pathway, drugs, prostitution and theft are framed as survival strategies. Looking at some of Brennan and colleagues [30] female pathways, burglary and drug trafficking are related to economic motives, whereas violent offences are linked to retaliation motives. However, the main focus of these studies is on traditional/gendered biographical elements contributing to these behaviours, not on the function or purpose of the behaviour. Profound insight into the specific functionality underlying DFA’s (re)offending is required in order to identify mechanisms of change and provide effective, tailored treatment [31]. More recently developed, holistic, strength-based rehabilitation frameworks can contribute to this matter by yielding new, aetiological insights, explicitly addressing the level of functionality.

### 1.2. Aetiological Relevance: Addressing the Level of Functionality

There is a recent, growing interest in the use of holistic strength-based rehabilitation frameworks, such as the Good Lives Model (GLM) [32], in guiding treatment of DFA [33]. The GLM is holistic in that it considers the whole person, beyond just focusing on their offending behaviour. It considers their wider goals and aspirations and the systems (e.g., family, friends, services) and resources (e.g., skills) they have around them to support them in achieving this. The GLM strives for the fulfilment of individuals’ basic human needs and aims to reduce the risk of reoffending. The GLM offers a strength-based alternative approach to the rehabilitation of DFA [32] and can help overcome the aetiological limitations of the RNR model [34]. More specifically, by tapping into DFA’s underlying basic human needs, the GLM can help provide more insights into the functionality of DFA’s behaviour. As such, the GLM may help with understanding how DFA became involved in offending and what helped them to find their way out of it.

### 1.3. Theoretical Application of the GLM’s Aetiological Assumptions to DFA

**Figure 1:**
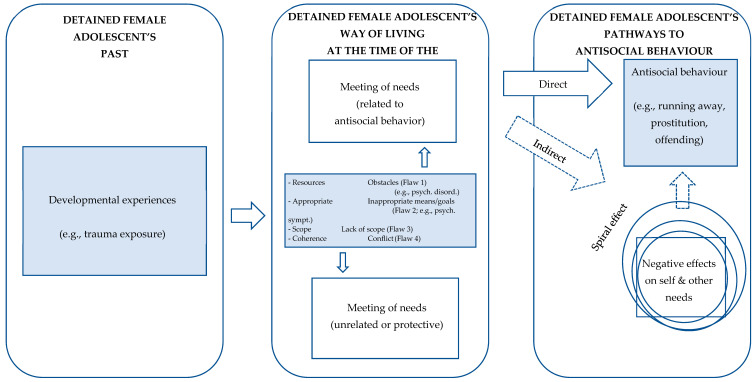
The Good Lives Model of offender rehabilitation applied to detained female adolescents (figure based on Van Damme et al., 2017; p.183). Note: Psych. sympt. = psychiatric symptoms; psych. disord. = psychiatric disorders. Figure based on the Goods Aetiological Theory [35] and the GLM-A [37].

The GLM was originally developed as a rehabilitation framework to explain sexual offending behaviour in adults [32]. Subsequently, the model has been applied to a broad range of offender populations [35], including youth offenders in general [36], and DFA in particular [34]. Underpinning the GLM is the assumption that humans all strive to achieve certain goals or values in life. These common, overarching goals are referred to as primary goods, or basic human needs and are the outcomes, states of being, or experiences that are valued by an individual. In line with Print’s (2013) adapted terminology of the GLM for use with young people, we use the terms “basic human needs”, “human needs”, or simply “needs”, instead of “primary goods” (see Table 1). Basic human needs contribute to an individual’s overall level of well-being, including their sense of happiness and fulfilment. According to the GLM, individuals all strive to fulfil the full range of 11 basic human needs, though may vary in which human needs they prioritise due to a range of factors such as individual differences in values, abilities, and life experiences [32]. According to the GLM, all people seek to achieve their personal goals through whatever means are available to them. The issue for some individuals, including offenders, arises when their efforts to achieve these goals are counter-productive, ineffective, and/or socially unacceptable, i.e., are illegal. Figure 1 illustrates the main assumptions, relating to DFA’s past, DFA’s way of living at the time of their antisocial behaviour, and pathways to antisocial behaviour. The GLM identifies four types of flaws (i.e., obstacles, inappropriate goals/means; lack of scope; and conflict) that can cause problems when individuals are trying to achieve their human needs. These are discussed in more detail below in relation to DFA.

The GLM can assist us in conceptualising and understanding which factors contributed to the development of antisocial behaviour (i.e., risk factors in an individual’s past), which factors contributed to the occurrence and maintenance of the antisocial behaviour, and how these factors come together in terms of understanding the individual’s overall pathway to antisocial behaviour. With regard to DFA’s past, it is assumed developmental experiences (e.g., poor attachment) affect DFA’s way of living and contribute to the development of antisocial behaviour [35]. With regard to DFA’s way of living at the time of the antisocial behaviour, the GLM identifies four types of flaws (i.e., obstacles, inappropriate goals/means; lack of scope; and conflict). Internal obstacles (e.g., poor self-regulation skills) and external obstacles (e.g., limited opportunities) are assumed to impede the achievement of DFA’s needs [32], in contrast to the use of inappropriate means/goals. In line with Print’s [38] adapted terminology of the GLM for use with young people, we use the term “means/goals”, instead of “secondary goods”. A secondary good typically takes the form of an approach goal first (e.g., an individual is looking for a job in order to achieve a sense of excellence in work). Once the individual has access to the good, it takes the form of a concrete mean [35] (e.g., dealing drugs in order to survive) and is assumed to hinder the realisation of needs when they turn out to be counter-productive [39]. *Lack of scope* refers to instances where DFA have a narrow focus on a very limited number of needs in their lives (e.g., prioritising pleasure and independence), thereby neglecting other needs [32]. In other words, DFA’s lives are considered to lack scope when they fail to secure (at some level) each of the 11 basic human needs [35]. With regard to conflicts, the GLM differentiates between horizontal conflicts (i.e., the lack of coherent relationships between or within different needs) and vertical conflicts (i.e., the lack of coherent ranking of different needs (e.g., attaching high importance to agency, but being required to obey your teacher or employer) [35,39]. An example of horizontal conflict is prioritising achieving inner peace through extensive substance use which negatively impacts your ability to achieve other needs such as physical health and excellence at school/work. An example of a vertical conflict is attaching high importance to agency, whilst being required to obey your teacher or employer. With regard to pathways to antisocial behaviour, the GLM assumes poor fulfilment of DFA’s needs enhances the risk of antisocial behaviour, with both direct and indirect pathways existing between poor fulfilment of needs and antisocial behaviour [32,35]. A direct pathway occurs when DFA actively attempt to secure their needs through antisocial behaviour (e.g., using violence to gain a sense of independence). An indirect pathway appears when poor fulfilment of DFA’s needs (e.g., trying to achieve inner peace by using drugs) leads to an accumulation of negative effects on their lives (e.g., problems at school), which finally leads to normative violations, for example, violent offending [35].

When applying the GLM’s aetiological assumptions to DFA from a theoretical perspective, it is of indisputable importance to consider the particular prevalence and nature of trauma exposure, psychiatric disorders, and antisocial behaviour in this population [8,11,12]. Figure 1 highlights how these gender issues can be integrated within a GLM framework. Trauma exposure can be considered as an important developmental experience, which is likely to influence DFA’s way of living and to instigate involvement in antisocial behaviour throughout their lives. Psychiatric disorders can be considered as internal obstacles that are likely to hamper the fulfilment of needs (e.g., PTSD may serve as an obstacle for achieving inner peace), whereas some psychiatric symptoms can be viewed as inappropriate attempts to fulfil one’s needs (e.g., aggressive behaviour may be displayed to achieve a sense of independence). As DFA’s engage in a wide range of antisocial behaviours it is useful to not limit the focus to officially recorded criminal behaviours but consider a broad range of behaviours that could negatively impact their and/or others’ safety and wellbeing [34].

### 1.4. Empirical Base of the GLM’s Aetiological Assumptions among DFA

To date, as far as we know, there are only two related empirical studies in which quality of life (QoL) was used as an indicator of the fulfilment of needs, in order to test the GLM among DFA [13,33]. The first study showed DFA were most satisfied with their social relationships and least satisfied with their psychological health prior to detention. Trauma exposure, psychiatric disorders, and a low socioeconomic status had a negative influence on multiple domains of DFA’s QoL prior to detention, which supported the GLM’s assumption regarding adverse developmental experiences, and internal and external obstacles impeding their ability to live a good life [13]. The second study provided partial support for the GLM’s assumptions regarding pathways to offending. An indirect pathway was found from QoL prior to detention via mental health problems to offending after discharge [33]. This means that DFA with a low QoL had an increased risk for mental health problems, which placed them at risk for offending subsequently. A direct negative pathway from low QoL prior to detention to increased offending after discharge was not found [33]. However, the study did reveal a direct positive pathway, with DFA who were highly satisfied with their social relationships prior to detention displaying increased offending after discharge. This is consistent with evidence that detained girls often affiliate with deviant peers [14], which can foster further engagement in criminal activities [21].

Although these two studies bolstered what is known about DFA’s fulfilment of needs, they do not provide profound insight into the complexity of the four types of flaws in their Good Lives plans (Figure 1). For example, evidence showed DFA were most satisfied with their social relationships (cf., the fulfilment of the need relatedness). Unfortunately, such evidence does not illuminate how the young women tried to fulfil this particular need (e.g., through involvement with deviant peers; cf., the use of inappropriate means; Flaw 2; Figure 1). In addition, these studies examined QoL by means of self-report questionnaires, which were characterised by a highly structured answering format and a priori defined life domains [13,33]. This study was the first to gain a deeper understanding of the functionality of DFA’s behaviour through the use of in-depth interviews with young women about their lives prior to and after detention.

The present qualitative study was designed to examine the functionality of young women’s behaviour prior to and four years after youth detention, using the GLM as the guiding theoretical framework. Young women’s life stories were explored, tapping into the fulfilment of their basic human needs prior to and after detention. That is, what function did their antisocial behaviour have in their efforts to fulfil their desired human needs? Important developmental experiences, flaws, and pathways to antisocial behaviour (Figure 1) were mapped. We also looked for turning points [26,40] that may have triggered positive changes in DFA’s behaviour over time.

## 2. Method

### 2.1. Setting

The study setting was an all-female YDC in Flanders, Belgium. Every year, about 140 female adolescents are placed in this YDC, for an average duration of 3 months [41,42]. Female adolescents are placed in a YDC by juvenile court judges due to criminal offending (e.g., shoplifting, fighting) or an urgent problematic educational situation (e.g., persistent truancy, prostitution). Placement in a YDC is considered the harshest measure a juvenile court judge can impose in Belgium. The YDC’s confining infrastructure and rigorous regime are intended to ensure a safe environment, whereas the treatment programme aims to promote adolescents’ resocialisation and reintegration [43].

### 2.2. Participants

The present study focused on the qualitative assessment interviews which occurred at 4 years after discharge from the YDC (T4). Participants were female adolescents who were admitted to the above-mentioned YDC between February 2012 and June 2014 (T0). T4 interviews were conducted between June 2017 and February 2018, about four years after DFA’s discharge from the YDC (T4; range: 3.02–5.28 years; M = 4.43; SD = 0.56). Saturation had been reached by the time we conducted 30 interviews, which meant continued data gathering was assumed not to reveal new themes [44]. In Text S1 in the Supplementary Materials, available online, we provide details regarding the baseline sample (T0; N = 147). Text S2, also available online, includes details regarding the longitudinal research design and the multiple waves of assessment (T0–T4). Details regarding differences between T4 participants (*n* = 30) and the remaining participants (*n* = 117) can be found in Text S3 and Table S1, both available online. At T4, participants were between 18.48 and 22.83 years old (M = 20.80; SD = 1.34), 27% attended education, 20% had a job, 80% had a partner, 33% had children, and 13% lived in a facility or centre (i.e., a residential facility for individuals with behavioural and/or emotional problems, or a centre providing temporary shelter and support for homeless people).

### 2.3. Procedure

In Text S4, available online, we provide details regarding the recruitment procedure of the baseline sample (T0). The qualitative follow-up assessment at T4 was conducted by the first author. The assessment took place at a time and place that were convenient for the participant. Interviews lasted between 30 min and 2 h and 37 min. All participants received a voucher for participating in the follow-up interview. The Ethical Commission of the Faculty of Psychology and Educational Sciences at Ghent University (2016/12) approved the study. In accordance with this approval, and in recognition of the vulnerability of our participants, the research protocol included obtaining written informed consent from each participant and confirming their agreement to take part prior to the start of each interview, making it clear participants could withdraw from the study at any time, and respecting their confidentiality by not including identifying information in any research outputs.

### 2.4. In-Depth Interviews

The first and second authors developed an interview protocol to assess participants’ Good Lives conceptualisations and to gain insight into the functionality of their behaviour (available upon request from the first author). The interview protocol was developed by drawing upon prior GLM assessment protocols [39,45,46], and in consultation with Tony Ward (i.e., the developer of the GLM). The interview consisted of three main parts, exploring participants’ current (part 1), past (i.e., prior to admission to the YDC; part 2) and future (part 3) lives. For the purpose of the present study only data pertaining to the periods prior to and after detention (cf., their current lives) were used.

To introduce the background and focus of the study, the interviewer started the interview by presenting participants with a list of the 11 “basic human needs” (referred to as “human needs” hereafter). Each part of the interview began with an open invitation to talk (e.g., “Tell me a bit about yourself and your life at the moment”). Next, building upon the particular story of the participant, the interviewer asked more detailed exploratory questions (e.g., “What do you [try to] do on a day-to-day or regular basis to achieve this?”, “What do you think helps/prevents you from achieving this in your life?”) in order to reach a deeper understanding of important developmental experiences, flaws, pathways to antisocial behaviour (Figure 1), and turning points. Throughout the interview, and at the end of each part, the interviewer used the list of needs to provide structure, to summarise, to allow an (interim) check of the interviewer’s interpretations, and to conclude.

### 2.5. Data Analysis

All interviews (*n* = 30) were transcribed verbatim and analysed by means of thematic analysis, a widely-disseminated analytic method that offers a robust and systematic framework for coding qualitative data [47]. Because this study aimed to examine and illustrate the GLM’s aetiological assumptions among former DFA, the thematic analysis was theory driven, which meant we used the GLM mapping table [45] and a GLM coding protocol (available upon request from the first author). Both tools enabled us to identify developmental experiences, flaws, pathways to antisocial behaviour, and turning points. In order to allow a stepwise process of analysis, the sample was split by means of a stratified sampling strategy [48]. Five stratification criteria were used that were likely to determine the overall structure of their lives (i.e., [not] attending education, [not] having a job, [not] having a partner, [not] having children, [not] living in a facility/centre). Based on these stratification criteria we had nine strata. For each stratum, the (biggest) group of cases, consisting of the most rich cases (*n* = 18; those with the fuller and more detailed descriptions of their lives), was included in the first phase of analysis, and the remaining cases (*n* = 12) were included in the second phase of analysis (Table S2, available online). The first phase of analysis (conducted by the first author) consisted of (i) coding the first 18 transcripts (i.e., by reading, rereading, and writing down thematic descriptive notes, using the coding protocol); (ii) developing 18 individual case formulations (i.e., structuring descriptive notes by means of the GLM mapping table); (iii) developing a visual representation of each case; and (iv) developing an intermediate master table (i.e., fine-tuning coding and identifying preliminary themes, in close collaboration with the second author). The second phase (conducted by the first author) consisted of (i) coding the remaining 12 transcripts (i.e., by reading, rereading, and writing down thematic descriptive notes, using the coding protocol); (ii) adding information from the additional transcripts to the intermediate master table; and (iii) developing the final master table (i.e., discussing the preliminary identified themes with the second author: identifying superordinate themes, (re)naming themes, and selecting key themes). The third and final phase consisted of a respondent validity check (i.e., allowing participants to critically reflect upon the findings). All the names used in this manuscript and Supplementary Materials are pseudonyms.

## 3. Results

The results come from the thematic analysis of 30 in-depth interviews which focused on the fulfilment of young women’s basic human needs prior to and four years after youth detention. This section first presents the results on the functionality of female adolescents’ behaviour prior to detention, then in relation to the functionality of their behaviour four years after detention. Results are presented with respect to the importance and content of the 11 human needs, and absence/presence of each of the flaws; internal/external resources/obstacles (Flaw 1), (in)appropriate means/goals used to attain each of the basic human needs (Flaw 2), the (lack of) scope (Flaw 3), and, finally, the existence of coherence/conflict between human needs (Flaw 4). The four types of flaws are used as the basis for the results section, with developmental experiences and pathways to antisocial behaviour integrated into the discussions of Flaw 1 and Flaw 2, respectively. Table S3 in the Supplementary Materials, available online, includes quotes (1–32) illustrating the results.

### 3.1. Functionality of Behaviour Prior to Detention

This section presents the results in relation to the functionality of the young women’s behaviour prior to detention.

### 3.2. Importance and Content Prior to Detention

In their past life stories, the basic human needs of independence (*n* = 24; Quote 1; Table S3 and relatedness (*n* = 24; Quote 2; Table S3) are most frequently rated as most important by the young women, followed by inner peace and belonging. Independence was mainly defined in terms of directing one’s own life (*n* = 27) and to a lesser extent in terms of standing on one’s own two feet (*n* = 8). As can be seen in Table 2, a number of the remaining human needs were given lower levels of priority (e.g., knowledge, excellence in school) in the lives of the young women prior to detention.

### 3.3. Internal/External Resources/Obstacles Prior to Detention (Flaw 1)

As can be seen in Table 3, the most prominent internal resources that appeared in the young women’s past life stories pertained to the human needs of independence and excellence in play, and to a lesser extent excellence in school/work. Smaller numbers of internal resources were identified in the human needs of relatedness, inner peace, knowledge, and creativity. No resources were noted in relation to the human needs of pleasure, physical health, belonging, or meaning in life. Some young women mentioned particular skills (e.g., Activities of Daily Living; ADL/interpersonal skills such as self-assertion; Quote 3; Table S3) and positive traits (e.g., being determined/tenacious), having a positive attitude, and interests that helped them achieve the human needs of independence, excellence in play and school/work. For example, some young women identified specific leisure interests (*n* = 7; Quote 4; Table S3) and skills (e.g., musical or dancing skills; *n* = 4) as aiding them in fulfilling the human need of excellence in play.

The young women identified noticeably more internal obstacles (see Table 4) to fulfilling their desired human needs prior to detention, than they did internal resources. The most prominent internal obstacles that appear in the young women’s past life stories related to the human needs of inner peace, excellence in school/work, physical health, independence, and relatedness. More than half of the young women mentioned how negative cognitions/affects (e.g., anxiety, anger, sadness; *n* = 18) hampered their peace of mind (Quote 5; Table S3), whereas about half of the young women referred to particular behavioural problems (e.g., truancy, running away; *n* = 13) which had a negative effect on their performance at school (Quote 6; Table S3). In addition, substance use problems (*n* = 10), a lack of ADL/interpersonal/emotion regulation skills (*n* = 9), and a lack of interpersonal skills (*n* = 8) seemed to challenge the achievement of physical health, independence, and relatedness, respectively.

The young women identified very few external resources in their past life stories (see Table 3) relating to many of the human needs. For example, the external resource of parental support was seen as enhancing their ability to achieve independence by a small number (*n* = 2). Here, good parenting could be perceived as a developmental experience, having a positive impact on the young women’s lives. As can be seen in Table 3, either very few or no external resources were identified by the young women as assisting them in achieving the other human needs prior to detention.

The young women identified numerous external obstacles in their past life stories (see Table 4) relating to achieving many of the human needs. The most prominent external obstacles in the young women’s past life stories related to the human needs of inner peace, relatedness, excellence in school/work and play, belonging, independence, and physical health. For example, the achievement of the human needs of relatedness (Quote 7; Table S3) was hampered by factors such as poor upbringing (*n* = 18), limited social support and/or rejection and bullying from family and/or friends (*n* = 12), and disconnection from social networks caused by repeated institutional placements (*n* = 7). Additionally, the young women’s sense of independence seems to have been mainly hampered by a limited freedom of choice in various ways (e.g., due to parents, the justice system, and placement in institutions with strict rules; *n* = 18; Quote 8; Table S3), but also other factors including poor upbringing (*n* = 8), poor living conditions (e.g., exposure to trauma and living in unsafe and unstable living conditions; *n* = 4), and poor social support from peers and family (*n* = 3). Here, poor upbringing and poor living conditions could be perceived as important developmental experiences, having a negative impact on the young women’s lives. The achievement of the other human needs was also all hampered by various obstacles (see Table 4). 

#### 3.3.1. (In)Appropriate Means/Goals and Their Relationship to Antisocial Behaviour Prior to Detention (Flaw 2)

As can be seen in Table 5, the most prominent appropriate means/goals that appeared in the young women’s past life stories referred to the human needs of inner peace, relatedness, and pleasure. For example, almost half of the young women mentioned the use of adaptive coping strategies (e.g., seeking professional, social or spiritual support, engaging in relaxing/distracting activities; *n* = 14; Quote 9; Table S3) in order to achieve a sense of inner peace. Other young women reported they (strived to) engage(d) in healthy relationships with family *(n* = 12), friends (*n* = 5), or others (e.g., caregivers; *n* = 6; Quote 10; Table S3) in order to fulfil their need of relatedness. Depending on the specific life story, the relationship of these appropriate means/goals to the young women’s past antisocial behaviour was either unrelated or protective. Smaller numbers of young women engaged in various appropriate approaches in an effort to achieve other human needs (e.g., physical health, excellence in play and school/work, knowledge, and creativity). The young women reported using no appropriate strategies to achieve the remaining human needs (e.g., independence). 

What stood out for this group of young women was the much greater numbers of inappropriate means, identified in their life stories prior to detention, used in an effort to achieve many of the human needs (Table 5). The most prominent inappropriate means related to the human needs of inner peace, independence, relatedness, belonging, and pleasure. The majority of young women mentioned the use of maladaptive strategies (e.g., substance use, running away, aggression, self-harm; *n* = 22; Quote 11; Table S3) in order to achieve inner peace. As can be seen in Table 5, substance use, a range of problematic behaviours (i.e., antisocial behaviour, deceitfulness, aggressive or threatening behaviours, truancy, running away, promiscuous sexual behaviours, etc.), and romantic relationships and friendships with antisocial individuals were all used by the young women as inappropriate strategies for obtaining/maintaining the other human needs, such as independence, relatedness, and pleasure. 

Depending on the specific life story, the relationship of these inappropriate means to achieving past antisocial behaviour was either unrelated, or directly or indirectly related. The indirect relationships were the result of the spiral effects that could be identified in eighteen of the life stories, with the majority being instigated by running away (n = 9; Quote 12; Table S3) or by an unhealthy romantic relationship (n = 5; Quote 13; Table S3). 

#### 3.3.2. (Lack of) Scope Prior to Detention (Flaw 3)

Three past life stories indicated adequate scope, which means DFA were securing (at some level) each of the 11 basic human needs. In contrast, for the majority of DFA, their past life stories indicated a lack of scope, indicated by a narrow focus on a very limited number of needs and the neglect of other needs (*n* = 19; Quote 14; Table S3). Human needs most frequently neglected were the primary goods of excellence in school/work (*n* = 18), excellence in play (*n* = 18), knowledge (*n* = 17), physical health (*n* = 17), meaning in life (*n* = 16), and creativity (*n* = 15). Their life stories also indicated other primary goods were neglected in the lives of some DFA prior to detention; inner peace (*n* = 10); belonging (*n* = 2); pleasures (*n* = 3); independence (*n* = 1).

#### 3.3.3. Coherence/Conflict Prior to Detention (Flaw 4)

The life stories of a small number of DFA had some level of conflict between particular human needs prior to detention (*n* = 6), whereas there were few indications of coherence, with one showing elements of conflict and one showing elements of both conflict and coherence. Issues with conflict with other human needs were particularly evident for the human needs of independence. For this human need, a vertical conflict was noted with the human goods of relatedness (*n* = 6; Quote 15; Table S3) and belonging (*n* = 2; Quote 16; Table S3). More specifically, the DFA put high value on their independence, while their lives were dominated by manipulation of a romantic partner (*n* = 5) and/or peer pressure (*n* = 1). Horizontal coherence was noted by a very small number of DFA between the human goods of inner peace and relatedness (*n* = 1), specifically in relation to their partner. Two DFA had been able to regain horizontal coherence between a sense of inner peace and their relationships with their parents (relatedness) prior to detention. More specifically, these DFA managed to regain a sense of peace of mind, by distancing themselves from their parents.

### 3.4. Functionality of Behaviour Four Years after Detention

Attention now turns to the results in relation to the functionality of the young women’s behaviour four years after detention. As with the previous results, this section first considers the importance and content of the 11 basic human needs (human needs) followed by each of the flaws. Additionally, this section considers turning points identified by the DFA in their life stories.

#### 3.4.1. Importance and Content after Detention

As seen in Table 2, the needs of relatedness (*n* = 29; Quote 17; Table S3), independence (*n* = 29; Quote 18; Table S3), and inner peace (*n* = 26; Quote 18; Table S3) were most frequently rated as most important, followed by excellence in school/work (*n* = 14), meaning in life (*n* = 14), and physical health (*n* = 13). The human needs were defined in relation to various content components. For example, independence was defined both in terms of directing one’s own life (*n* = 28) and standing on one’s own two feet (*n* = 27), and physical health consisted of three components, namely, healthy functioning (*n* = 30), financial and material conditions (*n* = 22), and safety (*n* = 2).

#### 3.4.2. Internal/External Resources/Obstacles after Detention (Flaw 1)

The most prominent internal resources that appeared in the young women’s current life stories (see Table 3) pertained to the needs of independence, inner peace, excellence in school/work, and relatedness. The majority of the young women mentioned particular skills (e.g., daily living, emotion regulation skills, interpersonal skills; Quote 19; Table S3) and positive personality traits (e.g., persistency, optimism; Quote 19; Table S3) which helped them to achieve a sense of independence, inner peace (Quote 20; Table S3), and relatedness. Finally, a large number of the young women referred to having broad/specific academic/vocational interests (*n* = 24) and positive personality traits (e.g., persistency, confidence; *n* = 19) and skills (e.g., academic, vocational and interpersonal skills; *n* = 15) that had a positive effect on their performance at school/work.

The most prominent internal obstacles (Flaw 1; see Table 4) that appeared in the young women’s current life stories related to the needs of physical health, inner peace, relatedness, and excellence in school/work. A wide range of physical health problems were mentioned (e.g., physical injuries, unhealthy bodyweight, sleeping problems; *n* = 24; Quote 21; Table S3) as obstacles for healthy functioning. In addition, negative cognitions/affect (e.g., anxiety, anger, shame; Quote 22; Table S3) and poor emotion regulation skills, poor interpersonal skills (e.g., difficulties to trust), and negative traits (e.g., stubbornness, insecurity) seemed to hamper the achievement of human needs such as relatedness, inner peace, and performance at school/work.

The most prominent external resources that appeared in the young women’s current life stories related to the needs of inner peace, independence, and physical health. As can be seen in Table 3, both social support (e.g., the presence/support from their partner/family (Quote 23; Table S3) and prior/current professional support (e.g., therapy) seemed to serve as external resources, enabling the young women to fulfil multiple human needs, including peace of mind, independence, and physical health.

The most prominent external obstacles (Flaw 1; see Table 4) that appeared in the young women’s current life stories pertained to the needs of relatedness, excellence in school/work, and inner peace. Here, the common external obstacles of poor upbringing and poor living conditions appeared as important developmental experiences, having a negative impact on the young women’s lives.

#### 3.4.3. (In) Appropriate Means/Goals and Their Relationship to Antisocial Behaviour after Detention (Flaw 2)

The most prominent appropriate means/goals that appeared in the young women’s current life stories pertained to the needs of inner peace, relatedness, independence, pleasure, physical health, and excellence in school/work. As seen in Table 4, all of the young women indicated they (strived to) use(d) adaptive coping strategies (e.g., engagement in relaxing/distracting activities; *n* = 30; Quote 24; Table S3) in order to fulfil the need of inner peace. The young women commonly reported using a variety of appropriate means to achieve a range of other human needs. For example, a very large group of young women also mentioned they (strived to) engage(d) in healthy and supportive relationships with family (*n* = 28; Quote 25; Table S3), friends (*n* = 25) or their partner (*n* = 25; Quote 25; Table S3) in order to fulfil their need of relatedness. An equally large group (aimed to) function(ed) and live(d) independently (e.g., making one’s on choices, having a driver’s license; *n* = 26), as a way of directing one’s life and standing on one’s own feet. Depending on the specific life story, the relationship of these appropriate means/goals to the young women’s current antisocial behaviour was either unrelated or protective.

The most prominent inappropriate means (Flaw 2; Table 5) to appear in the young women’s current life stories pertained to the needs of inner peace, independence, relatedness, and pleasure. About half of the young women indicated using maladaptive coping strategies (e.g., substance use, aggression; *n* = 16; Quote 26; Table S3) to gain a sense of inner peace. In addition, a small number of young women mentioned other means, such as the use of aggression to strive for justice or defend themselves, unhealthy romantic relationships, and substance use as inappropriate strategies to gain human needs including a sense of independence (Quote 27; Table S3), relatedness and pleasure. Depending on the specific life story, the relationship of these inappropriate means/goals to the young women’s current antisocial behaviour was either unrelated or directly related (cf., the direct pathway). No indirect pathway or spiral effects could be identified.

#### 3.4.4. (Lack of) Scope after Detention (Flaw 3)

For the majority of the young women (*n* = 26), their current life stories indicated an adequate scope, which means they were addressing a broad range of needs. Post detention, only four life stories indicated a lack of scope. Lack of scope in these few cases were associated with the neglect of the human goods of excellence in play (*n* = 4), creativity (*n* = 4), belonging (*n* = 3), excellence in school/work (*n* = 3), physical health (*n* = 2), knowledge (*n* = 2), pleasure (*n* = 2), meaning in life (*n* = 2), relatedness (*n* = 1), and independence (*n* = 1).

#### 3.4.5. Coherence/Conflict after Detention (Flaw 4)

The young women’s stories of their lives after detention contained more references to coherence between particular human basic needs (*n* = 23), compared to indications of conflict (*n* = 12). The most prominent example of coherence pertained to the horizontal congruence between inner peace and relatedness, experienced by more than half of the young women (*n* = 17; Quote 28; Table S3). More specifically, these young women were regaining or had already managed to regain a sense of peace of mind, by distancing themselves from particular destructive contacts (including parents/family, *n* = 14; partners, *n* = 4; friends, *n* = 2). In addition, a smaller group of young women experienced vertical congruence between independence vs. relatedness (*n* = 14; Quote 29; Table S3), belonging (*n* = 5), and pleasure (*n* = 1). More specifically, these young women were regaining or had already managed to regain a sense of independence, again by distancing themselves from particular destructive contacts. In this way their need for independence, which they valued highly, was no longer dominated by manipulation of a romantic partner and/or peer pressure. The most prominent conflicts that appeared in the young women’s current lives were three horizontal conflicts between relatedness vs. inner peace (*n* = 3), excellence in play (*n* = 3), and independence (*n* = 3), which were each experienced by a very small number of young women (*n* = 3). More specifically, these young women experienced a struggle between particular valuable relationships (e.g., parents/family, partner/child/pet) and the achievement of peace of mind (e.g., due to arguments), the achievement of excellence in play (e.g., due to limited time), and the achievement of independence (e.g., due to limited self-assertion). Other horizontal conflicts existed between inner peace and both excellence in work (*n* = 1) and knowledge (*n* = 1); relatedness and excellence in play (*n* = 1); excellence in school/work and both pleasure (*n* = 1) and relatedness (*n* = 2); creativity and relatedness (*n* = 2); and, finally, pleasure and relatedness (*n* = 1).

#### 3.4.6. Turning Points after Detention

The most prominent turning points (see Table 6) that appeared in the young women’s current life stories all related to the human needs of independence. Firstly, some internal resources seemed to serve as turning points. Most prominent were the increase in particular skills (e.g., increased emotion regulation and self-care; *n* = 17; Quote 30; Table S3), in addition to the increase in particular positive personality traits (e.g., increased optimism and persistency; *n* = 8). Secondly, some external resources served as turning points, including social support (e.g., from partner/family (in laws)/others; *n* = 11; Quote 31; Table S3) and prior professional support (e.g., therapy, time-out; *n* = 9). Finally, some appropriate means/goals seemed to serve as turning points. Most prominent were the choice to live one’s life differently and adopt a prosocial lifestyle (e.g., living a boring/clean/responsible life; *n* = 17; Quote 32; Table S3), the choice to take care of oneself (e.g., distancing themselves from destructive contacts; *n* = 11), and particular states of affairs which are related to the developmental period of emerging adulthood (e.g., criminal responsibility, motherhood; *n* = 8).

## 4. Discussion

The current study was designed to examine and illustrate the applicability of the GLM’s aetiological assumptions to young women who have previously been held in youth detention centres, by means of a thematic analysis of in-depth interviews with 30 young women who had spent time in a YDC. More specifically, the present study aimed to gain insight into the functionality of young women’s behaviour prior to and four years after youth detention, using the GLM as the guiding theoretical framework and building upon available evidence in female desistance literature. We first consider the key themes that emerged from the study, turning points identified in the young women’s life stories, and the clinical implications of the results, before considering the strengths, limitations, and future directions. Finally, some concluding comments are made.

### 4.1. Main Findings

Here we reflect upon the most prominent themes that emerged when looking at the young women’s lives from a GLM perspective, both prior to and four years after detention. These main findings are considered within a developmental context of adolescence and emerging adulthood. Within their life stories, the young women identified the basic human needs they valued the most. The human needs of relatedness and independence were prominent, both prior to and four years after detention, followed by inner peace, and, to a lesser extent, belonging. The fact that priority was given to the human need of independence is not surprising because one of the key transitions occurring during adolescence, into emerging adulthood, is that of the young person individuating from their family, particularly their parents, and establishing their own identity [49]. The fact relatedness and belonging were also relatively highly placed also fits because, during this period of adolescence and emerging adulthood, as individuals move away from their family, peers become increasingly influential [49] and young women start establishing romantic relationships and building their own lives [50]. The particular importance they attached to the need of inner peace likely reflects their efforts in starting to come to terms with their past, which were often characterised by adverse living conditions, including trauma exposure [4,5].

Although some internal and external resources were identified in the young women’s past life stories, overall, the young women identified notably more internal and external obstacles (Flaw 1; Figure 1) than they did internal and external resources in their lives prior to detention. These internal and external obstacles align with risk factors associated with antisocial behaviour in general (e.g., substance use problems, personality traits such as impulsivity, poor parent–child relationships, and low educational engagement) [21], and with female youth in particular (e.g., mental health issues and abuse experiences) [34,51]. This is problematic as it means these young women experienced multiple obstacles to, and had few resources to assist them in, achieving a life they would find fulfilling and personally meaningful prior to detention. Post detention, both internal/external resources and obstacles (Flaw 1; Figure 1) were highly prevalent in young women’s life stories. Compared to their past lives, there was an overall increase in internal and external resources, helping them to fulfil particular needs. For example, increased emotion regulation skills and social support helped them to achieve a sense of independence and inner peace. Of note, besides these resources, the majority of young women still encountered multiple obstacles with regard to particular needs. The complex interaction of (emerging) resources and (recurring) obstacles is characteristic of the challenging process of desistance [52,53], with DFA being particularly vulnerable to poor outcomes in early adulthood [1,2,5]. This is further discussed when addressing the issue of turning points below.

What stood out in the life stories of these young women, prior to detention, was the much greater numbers of inappropriate means, compared to appropriate means (Flaw 2; Figure 1), used in an effort to achieve many of their desired human needs. This is problematic, as the presence of multiple obstacles or risk factors, few resources or protective factors, and an overabundance of inappropriate strategies to achieve their life goals, leaves these young women at greater risk of ongoing antisocial behaviour and may mean desistance is less likely [21,36,54]. Compared to their past lives, we saw an overall increase in appropriate means and a decrease in inappropriate means to fulfil needs four years after detention. This positive development is likely to be related to the increase in both internal and external resources [32,35]. It heralds an increase in means that have a potentially protective relationship with the young women’s antisocial behaviour, and a decrease in means that have a potentially direct relationship with their antisocial behaviours. We consciously chose to use the word “potentially” in order to highlight the fact that the true impact of a specific (in)appropriate mean in relation to the reduction in antisocial behaviour always depends upon the specific context in which it occurs, including the other elements of the individual’s Good Lives plan (e.g., the importance attached to a particular need, the availability of resources/obstacles) [35,55]. 

The young women’s life stories indicated the majority lacked adequate scope in the goals they were striving to achieve in their lives prior to detention, with many human needs being neglected (Flaw 3; Figure 1). This is an issue because, according to the GLM, lack of scope can contribute to an imbalance where some areas of an individual’s life (e.g., employment, meaning in life) are underdeveloped [24]. An important positive development was that their post-detention live stories suggested they were embracing a much broader range of needs, resulting in a more balanced Good Lives plan.

Their life stories indicated only a small number of young women were experiencing conflict between particular human basic needs, referring to the lack of coherence between the human goods present in their lives prior to detention (Flaw 4; Figure 1). The rather low level of conflict experienced by these young women prior to detention may be accounted for by the overall lack of scope. That is, the fact the young women were limited in the number of human needs they were striving to achieve reduced the opportunity for conflict between human needs to occur. However, the fact they had a lack of scope is in itself problematic and indicates there were some human needs which they were not attending to at all in their lives. The conflict that existed between the various needs could cause tension in their lives and contribute to psychological distress and/or unhappiness [24]. The lack of coherence could contribute to an individual feeling frustrated and result in a life which lacks purpose or meaning [56]. Compared to the young women’s past life stories, there was an overall increase in coherence between different needs (Flaw 4; Figure 1) post detention, which is an important positive development. For example, more than half of the young women were regaining or had already managed to regain inner peace by distancing themselves from destructive contacts. However, almost half of the young women still encountered some form of conflict between particular needs, which is not surprising given the overall poor outcomes that are often reported for this particular group [1,2,5].

Overall, despite the fact that obstacles and conflict were still present in many of the young women’s post detention lives, we did see an important positive development, compared to their lives prior to detention. Throughout their post-detention life stories, the young women referred to a number of turning points they may have accounted for this development. These turning points were mostly related to the need of independence, which is not surprising as it reflected their need to build and direct their own lives [32]. The turning points appeared in different forms: not only as internal and external resources (e.g., increased emotion regulation skills and social support), but also as appropriate means (e.g., the choice to adopt a prosocial lifestyle). In line with the idea of desistance as a process, we also noted it was often not one single factor instigating a specific turning point [26,57]. Rather, multiple internal and external factors contributed to young women’s readiness for maintaining change, both initially and later on [52,53]. For example, the above-mentioned external resource of social support in itself may not be enough, because it should be complemented by young women’s emotional capacity to benefit from this support, in order to instigate true change [58].

### 4.2. Clinical and Policy Implications

Overall, the results of the present study provide a useful insight into the application of the GLM’s aetiological assumptions to young women who have spent time in a youth detention centre. The findings indicate the potential relevance of GLM-informed interventions with detained young women. Not only does this study provide us with additional insight into internal and external obstacles (which align with criminogenic needs) faced by young women, but indicates these obstacles should be considered in relation to the underlying needs they hamper/support, the accompanying (in)appropriate means/goals and their relationship to antisocial behaviour, and the issues of (lack of) scope and conflict/coherence. Doing so will give us deeper understanding of the functionality of their behaviour, which in turn can inform approaches to interventions [36]. A GLM-informed rehabilitation process consists of six phases, with Good Lives Assessment (phases one and two) being followed by Good Lives Planning (phases three to six) [39]. The planning phase should be about identifying programming which will assist them in achieving balance in their lives, and also provide them with the skills and supports necessary to achieve their life goals in adaptive and prosocial ways [36,59]. Failure to do so could result in these young women being at risk of continuing on an antisocial trajectory and struggling to find purpose in life and achieving a life which they find personally meaningful; see Van Damme et al. [34] for a more detailed description of these phases, including consideration of specific developmental and gender issues when applying it to this particular population.

Understanding the range of issues facing young women in detention settings is important and has implications for both policy makers and clinicians. The young women in this study presented with a wide range of risks and needs coming into detention. The results of this study suggest the focus of interventions could be broadened from a narrow focus on dynamic risk factors, to a more holistic approach, explicitly addressing the different GLM constructs. The underlying needs are the central theme throughout the process of rehabilitation because they appear to be important factors contributing to the occurrence of antisocial behaviour, and therefore are possible key motivational factors leading towards prosocial behaviour [16,35]. Overall, it is recommended that treatment programmes for justice-involved female adolescents consider including multidimensional, systemic, and gender-responsive components [60,61]. The current findings suggest public policy and interventions should address the young women’s multiple and interacting (criminogenic) needs, such as alcohol/drug use, mental health needs, and trauma exposure [62,63,64], and promote healthy connections with family, peers, school, and the wider community [65,66]. The gender-responsive component highlights the importance of policy makers to consider the potential need for a gender-specific approach and for programmes which address gender-specific (criminogenic) needs in a gender-responsive manner [11,12,22]. Policy makers and practitioners also need to be mindful of individual differences, such as ethnic origin, intellectual ability, sexual orientation, and pathways to offending [66,67,68].

Given the central role of the need of independence with regard to turning points in the young women’s life stories, our findings indicate youth detention centres could create a positive, open, and supportive living group climate to support opportunities for personal growth [69,70,71]. In other words, both policy makers and clinicians should consider making sure the support provided does not operate as an external obstacle to the young women meeting the need of independence (cf., the risk of institutionalisation, as indicated by some of the young women; Table 1) [58]. The focus on independence in the young women’s post-detention life stories challenges clinicians to adopt a collaborative rehabilitation approach, enabling the detained young women to be in charge of their own Good Lives plan [34]. Overall, these recommendations align well the GLM’s holistic and person-centred approach.

### 4.3. Strengths, Limitations and Recommendations for Future Research

This study has several strengths, including the focus on an often overlooked, yet clinically imperative, level of understanding (i.e., the level of functionality), and the use of an understudied but highly relevant population to examine and illustrate the GLM’s aetiological assumptions, further expanding the populations for whom this rehabilitation framework can be applied [56,72]. Nevertheless, the above results should be interpreted in the context of some limitations.

Despite these valuable contributions, it is important to bear in mind that the initial sample was gathered from a youth detention centre in Flanders, Belgium. Those followed up as part of this study were primarily of Belgium origin, with a complex range of mental health issues and low levels of assessed quality of life at baseline (Text S3 and Table S1, available online). Moreover, it is likely we did not reach the most vulnerable, marginalised, or antisocial young women, as they were unable to be contacted or refused to cooperate for their own reasons. These differences may create bias and may limit the generalisability of our findings. The young women were also asked to retrospectively reflect their life prior to detention—this was some time before the interview and their recall/perspective may have been affected by the intervening period. In addition, our findings may be biased (including absence or underrepresentation of specific themes, such as sexuality, gang membership) as a result of non-disclosure of particular information, for example, due to social desirability or trauma, or simply due to the snapshot nature of an interview. Therefore, future longitudinal studies are warranted to identify the extent to which our findings can be replicated in other, larger samples of detained young women.

Second, the qualitative method used here (i.e., theory-driven thematic analysis) provided insight into the most prominent themes and trends for the whole sample of detained young women, but did not help to elucidate how different needs interact and ultimately lead to the development of certain behaviours within individuals. Case study analyses are needed to yield more profound and contextualised insights into the complexity of young women’s behaviour prior to and after detention. This would allow for a richer and deeper understanding of the function of the behaviours in the young women’s lives which would, ultimately, also help inform intervention with young women in detention and other justice contexts. The findings could also be supported by future quantitative research to confirm the findings of this qualitative study.

## 5. Conclusions

This study is the first to explore the applicability of the GLM’s aetiological assumptions and provide a comprehensive overview of the functionality of young women’s behaviour prior to and after detention. Prior to detention it was clear that problems existed, with many of the young women lacking the internal and external resources (including prosocial and adaptive skills and supports) needed to attain their desired human goods, and often relying on antisocial and maladaptive strategies to achieve them. Some experienced conflict between the particular human needs they valued and wanted to achieve in their lives. That is, overall, their lives lacked a consistent and coherent approach to achieving what they wanted in life. Four years after detention, there was a trend towards improvement in all areas of functioning, with the young women having more balanced lives and increased internal and external resources to achieve the lives they wanted. The results have clinical implications, including highlighting the importance of developing a broad and in-depth understanding of the factors contributing to young women engaging in antisocial behaviour, in order to ensure appropriate interventions are put in place to assist these women in living a prosocial life that matters to them. The findings suggest a holistic, strength-based rehabilitation framework, such as the GLM, may help to enable a more effective and human approach to the rehabilitation of detained young women. The findings indicate there are opportunities for treatment to assist these young women, through the development of appropriate resources and, in some cases, alternative means to achieving their goals and in moving towards a personally meaningful and prosocial life.

## Figures and Tables

**Table 1:** The 11 basic human needs [32].

1	Physical health (i.e., being healthy and meeting basic needs)
2	Inner peace (i.e., feeling calm and relaxed and trying to reduce stress)
3	Relatedness (i.e., feeling connected to others through relationships with family, friends, romantic partners and other)
4	Belonging (i.e., belonging to social groups or groups with shared interests)
5	Excellence in school ^a^/work (i.e., being good at school/work)
6	Excellence in play (i.e., being good at and enjoying leisure or recreational activities)
7	Knowledge (i.e., having information and understanding about oneself and the world)
8	Creativity (i.e., having novelty or innovation in one’s life)
9	Pleasure (i.e., feeling content or experiencing pleasure)
10	Independence (i.e., directing one’s own life)
11	Meaning in life (i.e., finding meaning or a purpose in life)

^a^ School was added in order to fit within the development period of adolescence.

**Table 2:** Importance and content identified in the young women’s life stories prior to and after detention.

	Prior to Detention		After Detention	
Basic Need	Importance (*n*)	Content (*n*)	Importance (*n*)	Content (*n*)
Physical health	Low (19) Moderate (0) High (4)	Safety (2) Healthy functioning (20) Financial & material conditions (incl. stability; 3)	Low (2) Moderate (15) High (13)	Safety (2) Healthy functioning (30) Financial/material conditions (22)
Inner peace	Low (10) Moderate (1) High (16)		Low (0) Moderate (4) High (26)	
Relatedness	Low (0) Moderate (2) High (24)		Low (1) Moderate (0) High (29)	
Belonging	Low (3) Moderate (5) High (15)		Low (15) Moderate (11) High (3)	
Excellence in school/work	Low (23) Moderate (1) High (1)		Low (6) Moderate (10) High (14)	
Excellence in play	Low (17) Moderate (3) High (3)	Enjoyment (8) Being good at (5)	Low (9) Moderate (13) High (8)	Enjoyment (20) Being good at (15)
Knowledge	Low (20) Moderate (2) High (1)	Knowledge about the world (3) Knowledge about oneself (0)	Low (13) Moderate (10) High (5)	Knowledge about the world (21) Knowledge about oneself (8)
Creativity	Low (0) Moderate (2) High (3)	Creating things (3) Novelty seeking (4)	Low (16) Moderate (8) High (6)	Creating things (15) Novelty seeking (0)
Pleasure	Low (3) Moderate (10) High (9)	Fun & enjoyment (14) Kicks & excitement (9)	Low (2) Moderate (20) High (8)	Fun & enjoyment (29) Kicks & excitement (0)
Independence	Low (2) Moderate (2) High (24)	Directing one’s own life (27) Standing on one’s own feet (8)	Low (0) Moderate (1) High (29)	Directing one’s own life (28) Standing on one’s own feet (27)
Meaning in life	Low (19) Moderate (1) High (2)	Broader meaning (2) Religious/spiritual meaning (1)	Low (8) Moderate (8) High (14)	Broader meaning (19) Religious/spiritual meaning (4)

**Table 3:** Internal and external resources identified in the young women’s life stories prior to and after detention.

	Internal Resources		External Resources	
Basic Need	Prior to Detention (*n*)	After Detention (*n*)	Prior to Detention (*n*)	After Detention (*n*)
Physical health		Skills (4) - daily living skills		Professional support (10) - learned daily living skills in YDC/therapy, current professional support (e.g., housing, medical)
		Positive personality traits (3) - persistency, optimism		Social support (7) - support from partner/family/friends (e.g., practical, financial)
				Good living conditions (5) - safe/stable/comfortable living conditions
Inner peace	Skills (2) - self-care	Skills (24) - self-care, personal growth (e.g., increased emotion regulation skills)		Social network/support (18) - presence & support from partner/child/friends/family (in law)/pets/other
		Positive personality traits (6) - optimism, persistency		Professional support (11) - learned emotion regulation skills & experienced support in/from YDC/psychiatry/lawyer/other, current professional support from psychologist/caregiver
		Recovery (4) - recovery from alcohol/drug/mental health problems		Good upbringing (3) - good parenting
		Positive attitudes (2) - openness for help/support		
Relatedness	Positive traits (2) - confidence and extroversion	Skills (21) - interpersonal skills, self-care Positive personality traits (9) - extroversion, persistency, optimism		Social network/support (7) - presence & support of partner/friends/family
Belonging		Positive personality traits (2) - likeability, humour	Social inclusion/network/opportunities/support (2) - connection with peers in the institution	Social inclusion/opportunities (3) - connection with peers, living conditions (institution) creating social opportunities
		Skills (2) - interpersonal skills		
Excellence in school/work	Interests (2) - specific academic interests	Interests (24) - academic/vocational interests.		Good educational opportunities/experiences (6) - former/current educational opportunities or positive experience (e.g., internship, adult education).
	Skills (4) - overall/specific academic skills and intellectual ability.	Skills (15) - academic, vocational, interpersonal skills.		Good job opportunities/experiences (6) - former/current job opportunities or positive experiences.
		Positive personality traits (19) - persistency, optimism.		Social support (6) - support from partner/grandparent/colleagues.
		Positive attitudes (6) - eagerness to learn, openness.		Professional support (4) - financial & practical support.
				Positive work environment (2) - nice colleagues.
Excellence in play	Interests (7) - specific leisure interests.	Interests (23) - leisure interests.	Leisure opportunities (2) - enforcement by judge to engage, utilising leisure opportunities in institutions	Leisure opportunities (4) - former opportunities to engage in leisure activities & learn leisure skills in YDC/in other institutions/at school, freedom to engage in activities of one’s own choice
	Skills (4) - specific leisure skills, incl. dancing, music	Skills (11) - leisure skills		
		Positive personality traits (4) - persistency, optimism		
Knowledge	Interests (2) - broad/specific interests.	Interests (15) - broad/specific interests		
		Skills (6) - personal growth (e.g., learned from the past), intellectual ability, interpersonal skills.		
		Positive attitude (4) - eagerness to learn, openness		
Creativity	Interests (3) - specific creative interests	Interests (13) - creative interests.		Leisure opportunities (2) - former opportunities to engage in artistic activities & learn creative skills in YDC/psychiatry/other institutions
		Skills (6) - creativity.		
Pleasure		Positive attitudes (4) - carpe diem attitude.		Social network/opportunities (6) - presence from partner/friends/family/pets, living conditions (institution) creating opportunities of social involvement & fun
		Positive personality traits (3) - persistency, optimism, humour		
Independence	Skills (7) - daily living skills - interpersonal skills.	Skills (26) - daily living skills, personal growth (e.g., increased emotion regulation skills), interpersonal skills, self-care.	Good upbringing (2) - good parenting, incl. parental support (2)	Good upbringing (2) - good parenting.
	Positive traits (4) - determined/tenacious.	Positive personality traits (20) - persistency, optimism.		Professional support (17) - learned emotion regulation/interpersonal/daily living skills & experienced support in YDC/psychiatry/other, current professional support from (former) caregiver/therapist/judge/other.
	Positive attitudes (2) - disapproving attitude towards drugs.	Positive attitude (6) - openness for help/support, conformism.		Social network/support (15) - presence & support from partner/family (in law)/others.
		Positive cognitions/affect (9) - feelings of happiness (e.g., pride, gratefulness).		Freedom of choice (11) - freedom of choice no longer restricted by (grand)parents/(youth)court.
		Recovery (3) - recovery from drug problems.		Social deprivation (6) - former isolation from antisocial peers & substances during YDC/time-out abroad, former experience/threat of deprivation during YDC/adult imprisonment.
Meaning in life		Skills (8) - personal growth (e.g., expert by experience).		Religious upbringing (2) - religious beliefs.
		Positive cognitions/affect (7) - feelings of happiness (e.g., satisfaction, pride).		Social network (2) - relationship with catholic partner, being surrounded by people who truly care.
		Positive personality traits (6) - persistency, optimism.		

**Table 4:** Flaw 1: Internal and external obstacles identified in the young women’s life stories prior to and after detention.

	Internal Obstacles		External Obstacles	
Basic Need	Prior to Detention (*n*)	After Detention (*n*)	Prior to Detention (*n*)	After Detention (*n*)
Physical health	Physical health problems (3) - unhealthy bodyweight, asthma	Physical health problems (24) - physical complaints, unhealthy bodyweight, sleeping problems.	Poor living conditions (10) - unhealthy living conditions, incl. trauma exposure - unsafe living conditions - unstable living conditions	Poor living conditions (12) - former unhealthy living conditions (e.g., trauma exposure), current unstable/uncomfortable living conditions (e.g., housing & financial problems)
	Substance use problem (10) - nicotine, drug & alcohol abuse/addiction.	Substance use problems (8) - nicotine/drug/alcohol abuse/addiction.		
	Behavioural problems/strategies (5) - self-harm, unhealthy food habits, aggressive behaviour.	Negative personality traits (5) - nonchalance, prodigality, laziness.		
Inner peace	Negative cognitive/affect (18) - feelings of anxiety, anger, sadness, loneliness, shame, identity issues, suicidal thoughts, preoccupation with loss	Negative cognitions/affect (23) - (social) anxiety, anger, sadness	Lack of (tailored) professional support (14) - (repeated) placement in institutions, incl. poor match with needs - negative experience with police officers	Lack of (tailored) professional support (9) - negative experiences with support from YDC/psychiatrists/judge/other, limited access to professional.
	Lack of skills (6) - poor emotion regulation skills	Lack of skills (12) - poor emotion regulation skills	Poor living conditions (13) - unhealthy living conditions incl. trauma exposure - unstable living conditions	Poor living conditions (15) - former unhealthy living conditions (e.g., trauma exposure), current unstable living conditions (e.g., limited financial resources)
	Substance use problem (2) - drug addiction	Substance use problems (3) - drug abuse/addiction	Poor upbringing (8) - poor relationships, incl. poor attachment - poor parenting, incl. lack of parental support	Poor upbringing (8) - poor relationship with parents, poor parenting
		Negative attitudes (5) - no openness for help/support	Loss (7) - death of a loved one (incl. abortion, miscarriages)	Loss (13) - death of a loved one, out-of-home placement of child
		Negative personality traits (3) - stress-sensitivity, hyper-sensitivity, insecurity	Lack of social inclusion/(3) - bullying by peers/rejection by family/peers	Lack of social inclusion/support (6) - limited social support, lack of appreciation from family, lack of connection with peers
			Injustice (3) - experiences of personal (placement) & social (unequal chances in life) injustices	Injustice (11) - experiences of personal & social injustice
			Life related conflicts/stress (4) - family related: incl. being searched by family - justice related: being searched by police	Life related stress (15) - family/partner/child/peer/justice related problems
				Stigma/judgment (4) - stigma regarding antisocial behaviour & drug addiction, judgment about motherhood
				Social deprivation (3) - former experience of social deprivation/isolation/fixation in YDC/psychiatry
Relatedness	Lack of skills (8) - lack of interpersonal skills	Lack of skills (12) - limited interpersonal skills (e.g., difficulties to trust).	Poor upbringing (18) - poor relationship with parent/s, incl.	Poor upbringing (23) - poor relationship with parents, poor parenting.
	Negative traits (5) - impulsive, naïve, stubborn	Negative personality traits (10) - stubbornness, dominance, insecurity	poor attachment (14) - poor parenting, incl. lack of parental support	Lack of social inclusion/network/support (11) - lack of social support & appreciation from partner/family, rejection by family, limited number of friends.
	Behavioural problems/strategies (2) - lying, deceiving, running away	Behavioural problems/strategies (2) - psychiatric problems (e.g., aggression)	Lack of social inclusion/network/opportunities/support (12) - limited support/appreciation from family/peers, few friends, bullying (by peers), social rejection	Poor living conditions (5) - former unhealthy living conditions (e.g., trauma exposure), current unhealthy/unsafe living conditions (e.g., antisocial neighbourhood).
		Substance use problems (2) - drug abuse/addiction	Poor living conditions (2) - unhealthy living conditions, incl. trauma exposure	Social deprivation (2) - former disruption of family/peer contacts due to (repeated) placement in institutions.
			Social deprivation (7) - lack of stable relationships with family/friends due to (repeated) placement in institutions	Life related stress (15) - family/peer related problems.
				Loss (8) - death of a loved one, out-of-home placement of child.
				Judgment (3) - judgment about motherhood/choice of partner.
				Practical barriers (3) - distance, time.
Belonging			Lack of social inclusion/network/opportunities/support (4) - lack of popularity/acceptance, bullying by peers.	Lack of social inclusion (2) - lack of connection with peers/others
			Social deprivation (2) - lack of a steady peer group due to repeated placements.	
Excellence in school/work	Negative cognitions/affect (6) -not feeling comfortable at school, performance anxiety, preoccupation with other things (e.g., running away).	Negative cognitions/affect (10) - (social/performance) anxiety, depression, preoccupation with other things.	Limited educational opportunities (6) - interrupted school.	Poor educational opportunities/experiences (20) - former poor educational opportunities/experiences (e.g., interrupted school career, no secondary education degree: e.g., due to placement in institutions).
	Negative attitudes (6) - rebellious, arrogant.	Negative attitudes (2) - anarchism.	Poor living conditions (2) - unhealthy living conditions, incl. sexual exploitation - unstable living conditions, incl. limited financial resources.	Poor living conditions (4) - unstable living conditions (e.g., limited financial resources).
	Lack of interests (5) - lack of specific academic interests.	Lack of interests (3) - lack of specific academic/vocational interests.	Lack of social inclusion/network/opportunities/support (7) - lack of connection with peers at school, bullying.	Practical barriers (8) - time, distance, presence of child.
	Substance use problem (2) - drug addiction.	Substance use problems (2) - drug/alcohol abuse/addiction.	Life related conflicts/stress (2) - family, justice and peer related.	Poor job opportunities/experiences (5) - limited (long-term) job opportunities, negative solicitation experiences, unemployment.
	Behavioural problems/strategies (13) - truancy, running away.	Lack of skills (8) - limited academic, interpersonal, emotion regulation skills.		Stigma/discrimination (4) - discrimination at the job market, stigma regarding not having a secondary education degree.
		Physical health problems (6) - phys. Complaints.		Poor upbringing (2) - poor parenting.
Excellence in play	Behavioural problem/strategies (2) - running away	Physical health problems (5) - physical complaints, unhealthy bodyweight.	Poor living conditions (2) - unhealthy living conditions, including sexual exploitation and unstable living conditions (incl. limited financial resources).	Poor living conditions (7) - unstable living conditions: (e.g., limited financial resources).
		Negative cognitions/affect (5) - sadness, (social) anxiety, depression.	Limited leisure opportunities (4) - limited opportunities to engage in leisure activities.	Practical barriers (6) - time, distance, presence of child/pet.
		Lack of interests (2) - lack of specific leisure interests.	Life related conflicts/stress (2) - family and justice related.	Lack of social network (2) - lack of company.
Knowledge		Lack of skills (2) - limited academic skills, limited intellectual ability		Poor educational opportunities/experiences (2) - former poor educational opportunities/experiences (e.g., interrupted school career, no secondary education degree: due to placement in institutions)
Creativity	Lack of skills (2) - limited creativity	Lack of skills (7) - limited creativity.		Poor living conditions (3) - unstable living conditions (e.g., limited financial resources).
		Interests (5) - limited creative interests.		Practical barriers (2) - time.
Pleasure	Negative cognitions/affect (3) - feelings of anxiety/stress (incl. hypervigilance), ruminating.	Negative cognitions/affect (8) - sadness, (social) anxiety, preoccupation with past/loss.	Poor living conditions (2) - unhealthy living conditions at home, including trauma exposure	Poor living conditions (5) - former unhealthy living conditions (e.g., trauma exposure), current unstable living conditions (e.g., limited financial resources).
		Physical health problems (2) - phys. complaints, sleeping problems.		Loss (4) - death of a loved one
Independence	Lack of skills (9) - limited daily living, interpersonal and emotion regulation skills.	Lack of skills (8) - limited daily living/interpersonal/emotion regulation skills	Poor living conditions (4) - unhealthy living conditions.	Poor living conditions (8) - former unhealthy living conditions (e.g., trauma exposure, manipulation by partner), current unhealthy/unsafe/unstable living conditions (e.g., antisocial neighbourhood, limited financial resources).
	Negative traits (4) - impulsivity, naivety, limited responsibility, immaturity, insecurity, lazy.	Negative personality traits (6) - nonchalance, impulsivity, insecurity	Limited freedom of choice (18) - freedom of choice restricted by parents, youth court, placements in institutions with strict rules.	Limited freedom of choice (8) - freedom of choice restricted by parents/court/broader societal norms.
	Negative cognitions/affect (4) - feelings of sadness, anger, identity issues.	Negative cognitions/affect (6) - sadness, anxiety, psychiatric problems (e.g., borderline).	Poor upbringing (8) - poor relationship/s with parent/s and poor parenting.	Poor upbringing (5) - poor relationship with parents, poor parenting.
		Vulnerabilities (9) - antisocial history/habits/tendencies.	Life related conflicts/stress (2) - peer related.	Life related stress (3) -/partner/peer related problems.
		Physical health problems (2) - phys. Complaints.	Injustice (3) -experiences of personal injustice (placement, not being heard/understood).	Injustice (5) - experiences of personal & social injustice.
			Lack of social inclusion/network/opportunities/support (3) - bullying by peers/rejection by family.	Institutionalisation (4) - difficulties to organise one’s life outside the context of an institution.
Meaning in life		Behavioural problems/strategies (5) - living from day to day.		
		Negative cognitions/affect (3) - feelings of sadness, suicidal thoughts, preoccupation with loss.		

**Table 5:** Appropriate and inappropriate (Flaw 2) means identified in the young women’s life stories prior to and after detention.

Basic Need	Prior to Detention (*n*)	After Detention (*n*)	Prior to Detention (*n*)	After Detention (*n*)
Physical health	Activities with health purposes (3) - taking care of oneself: adopting a healthy lifestyle.	Activities with health purposes (23) - taking care of oneself: adopting a healthy lifestyle.	Activities with financial/material purposes (2) - property & violent offending and drug dealing.	-
		Activities with financial/material purposes (17) - improving living conditions (e.g., getting/having a job).		
Inner peace	Adaptive coping (14) - seeking support, engagement in relaxing/distracting activities (e.g., writing, art, music, sports), living from day to day	Adaptive coping strategies (30) - engagement in relaxing/distracting activities, seeking (social/professional/supernatural) support, distancing oneself from destructive contacts	Maladaptive coping (22) - drug & alcohol use, - running away, physical/verbal aggression, self-harm, suicide attempt, rebellious behaviour	Maladaptive coping strategies (16) - drug use, material/verbal/physical aggression, isolating oneself
Relatedness	Family relationships (12) - with mother, father, other family (incl. siblings, grandparents, nephews/nieces) and family in law.	Family relationships (28) - mother, father, child, other family (in law).	Obtaining/maintaining relationships (11) - running away, firesetting, truancy & disobedience at home, promiscuous behaviour and drug use.	Obtaining/maintaining relationships (3) - sacrificing oneself, lying.
	Obtaining/maintaining relationships (2) - attending religious courses - making effort to be perceived positively by teachers.	Obtaining/maintaining relationships (4) - distance oneself from destructive contacts.	Romantic relationships (10) - unhealthy/unbalanced relationship with antisocial partner and frequently changing romantic relationships.	Romantic relationships (3) - unhealthy/unbalanced relationship.
	Friendships (5) - close friends.	Friendships (25) - close friends.	Friendships (5) - relationships with antisocial friends.	
	Other relationships (6) - with caregivers, peers/teachers at school and animals.	Other relationships (15) - (ex-)caregiver, neighbour, pet		
		Romantic relationships (25) - healthy/balanced relationship.		
Belonging	Being part (3) - being part of a group in the institution/at school.	Being part (7) - being part of a prosocial peer group (at school/work/other).	Being part (14) - hanging around with antisocial peers/people and gang membership.	Being part (2) - being part of an antisocial peer group/gang
		Becoming/staying part (4) - making efforts to fit in, engaging in voluntary work.	Becoming/staying part (14) - drug & alcohol misuse, rebellious behaviour, running away, truancy, property offending (incl. shoplifting) and physical aggression.	
Excellence in school/work	-	Work-related activities (22) - getting/having a meaningful job.	-	-
		School-related activities (14) - attending school (in order to get a degree).		
Excellence in play	Engagement in prosocial leisure activities (5) - engagement in music, sports	Engagement in prosocial leisure activities (17) - sports, music, acting, etc.	-	-
Knowledge	(In)formal learning (3) - engagement in courses, experience-based activities	(In)formal learning (19) - engagement in courses, informative activities, experience-based activities.	-	Personal growth (2) - engagement in antisocial mind-expanding activities (e.g., drug use)
		Personal growth (6) - engagement in prosocial activities of personal growth (e.g., therapy, self-reflection).		
Creativity	Engagement in artistic activities (3) - tinkering, drawing, dancing, music	Engagement in artistic activities (14) - tinkering, decorating, hairdressing	Engagement in experimental activities (3) - experimenting with drugs (incl. addiction)	-
Pleasure	Social involvement (4) - spending time with friends.	Social involvement (25) - spending time with child/partner/family (in law)/friends/pets.	Fun/exciting activities (15) - running away, drug & alcohol use, rebellious behaviour, partying, and property offending.	Fun/exciting activities (3) - drug/alcohol use
	Fun/exciting activities (6) - engagement in enjoyable activities (incl. watching a movie, reading, playing music, going on school trips	Fun/exciting activities (23) - engagement in fun activities (e.g., watching a movie, going out).	Social involvement (8) - spending time with antisocial peers/people.	
		Self-caring activities (7) - taking care of oneself, enjoying the small things in life	Self-boosting activities (4) - acting tough, drug dealing, physical aggression.	
Independence	-	Being/living independently (26) - making one’s own choices, getting/having a degree/job/driver’s license/car/own house.	Adopting an antisocial lifestyle (5) - homelessness, drug using or dealing.	Adopting a pro-/anti-social lifestyle (2) - being against ‘the system’.
		Adopting a pro-/anti-social lifestyle (18) - choosing to live one’s life differently.	Doing whatever one wants (21) - running away, rebellious behaviour, drug & alcohol use, partying, socialising with older/antisocial people, lying & deceiving, truancy, and firesetting.	Striving for justice/defending oneself (6) - acting tough, using verbal/physical aggression.
		Taking care of oneself (13) - distancing oneself from destructive contacts.	Obtaining status (19) - through physical, verbal, material aggression, through acting tough, rude, arrogant, aggressive, arguing with & contradicting other, property offending, threatening, bullying others and taking leadership position in antisocial peer group.	
		State of affairs related to emerging adulthood (9) - motherhood, having reached the age of legal majority & criminal responsibility.		
		Striving for justice/defending oneself (6) - defending one’s own rights/communicating one’s own boundaries with words.		
		Obtaining status/being someone (3) - living a luxurious life, being loud.		
Meaning in life	-	Broader personal meaning-making (14) - doing it better compared to one’s own parents, chasing one’s vocational dream.	Broader personal meaning-making (2) - looking for happiness/a better future via inappropriate means, incl. running away	-
		Broader altruistic meaning-making (14) - partnership/motherhood, giving one’s children a better future, helping others based on one’s own experiences.		
		Religious/spiritual engagement (4) - praying, immersing oneself in religious/spiritual/supernatural ideas.		

**Table 6:** Turning points identified in the young women’s life stories after detention.

Basic Need *	After Detention (*n*)
Inner peace	Internal resources Skills (7) Positive personality traits (2) Recovery (1)
	External resources Social network/support (5) Professional support (4) Good upbringing (1)
	Means/goals Adaptive coping strategies (4) Maladaptive coping strategies (1)
Relatedness	Internal resources Skills (1)
	External resources Social support (1)
	Means/goals Family relationships (3) Obtaining/maintaining relationships (2)
Belonging	Internal resources Positive personality traits (1)
	External resources Good educational opportunities/experiences (1)
Independence	Internal resources Skill (17) Positive personality traits (8) Positive cognitions/affect (1) Positive attitudes (3) Recovery (2)
	External resources Professional support (9) Social network/support (11) Freedom of choice (5) Social deprivation (6) Good upbringing (1)
	Means/goals Being/living independently (7) Adopting a pro-/anti-social lifestyle (17) Taking care of oneself (11) State of affairs related to emerging adulthood (8)
Meaning in life	Internal resources Skills (1) Positive personality traits (1)
	Means/goals Broader personal meaning-making (5) Broader altruistic meaning-making (4)

* Only those needs for which there were turning points are included in the table.

## Data Availability

The dataset generated and analysed during the current study are not publicly available due to confidentiality reasons, but are available from the corresponding author on reasonable request.

## Supplementary Materials

The following are available online at https://www.mdpi.com/article/10.3390/ijerph182211830/s1, alongside the manuscript: (a) Supplementary text: Text S1: Recruited participants of the baseline sample (T0); Text S2: Longitudinal research design (T0–T4); Text S3: Differences between T4 (*n* = 30) and remaining (*n* = 117) participants; Text S4: Recruitment procedure of the baseline sample (T0); (b) Supplementary tables: Table S1: Differences between T4 (*n* = 30) and remaining (*n* = 117) participants; Table S2: Stratified sampling strategy; Table S3: Overview of quotes, illustrating the results on the functionality of young women’s behaviour prior to and four years after youth detention.
