# Supplementary material for: A Qualitative Study on Young Women’s Lives Prior to and Four Years after Youth Detention: Examining the Good Lives Model’s Aetiological Assumptions
Source: Int J Environ Res Public Health. 2021 Nov 11;18(22):11830. doi: 10.3390/ijerph182211830 (PMC8625703; doi:10.3390/ijerph182211830)
Supplement: Supplementary file 1 [file ijerph-18-11830-s001.zip › 1. Supplementary tables.pdf]

International Journal of
*Environmental Research and Public Health*

MDPI

# Supplementary tables

Table S1

*Differences between T4 (n = 30) and remaining (n = 117) participants*

| | T4 participants *(n = 30)* | Remaining participants *(n = 117)* | T4 vs. remaining |
|---|---|---|---|
| | *M (SD)* | *M (SD)* | *t (df)* |
| Age | 15.92 (1.10) | 16.30 (1.08) | 1.69 (145) |
| QoL Physical health | 57.36 (14.54) | 65.93 (16.02) | 2.66 (145)** |
| QoL Psychological health | 44.17 (21.01) | 57.40 (20.25) | 3.17 (145)** |
| QoL Social relationships | 70.28 (20.84) | 78.23 (19.06) | 2.00 (144)* |
| QoL Environment | 58.44 (13.93) | 65.91 (18.61) | 2.05 (145)* |
| Non-violent offending | 4.80 (4.52) | 3.73 (3.78) | -1.32 (142) |
| Violent offending | 1.57 (1.83) | .99 (1.29) | -1.62 (36.74) |
| | *n* (%) | *n* (%) | $\chi^2$ (*df* = 1) |
| Origin (Belgian) | 27 (90.0) | 69 (59.0) | 10.14** |
| Socioeconomic status (low) | 17 (56.7) | 58 (56.9) | < .01 |
| Intact family (yes) | 3 (10.0) | 17 (14.7) | .44 |
| School attendance (yes) | 16 (53.3) | 70 (59.8) | .42 |
| Past detention history (yes) | 7 (23.3) | 23 (19.7) | .20 |
| Any psychiatric disorder (yes) | 28 (93.3) | 97 (85.8) | 1.21 |
| Any internalizing disorder (yes) | 24 (80.0) | 52 (44.8) | 11.82** |
| Any externalizing disorder (yes) | 25 (83.3) | 87 (76.3) | .68 |
| Pure internalizing disorder (yes) | 3 (10.0) | 11 (9.7) | < .01 |
| Pure externalizing disorder (yes) | 4 (13.3) | 46 (40.7) | 7.81** |
| Any comorbidity (yes) | 26 (86.7) | 67 (59.3) | 7.81** |
| Comorbidity in- and externalizing disorders (yes) | 21 (70.0) | 40 (35.4) | 11.60** |

*Note.* T4 = assessment four years after discharge; QoL = Quality of Life.

* $p < .05$; ** $p < .01$.

Table S2

*Stratified sampling strategy*

| | Stratification criteria | | | | |
|---|---|---|---|---|---|
| | Attending education | Having a job | Having a partner | Having children | Living in a facility/centre |
| Strata. (number of cases: total; first/second phase[a]) | | | | | |
| 1. (*n* = 7; *n* = 4/3) | no | no | yes | yes | no |
| 2. (*n* = 7; *n* = 4/3) | no | no | yes | no | no |
| 3. (*n* = 4; *n* = 2/2) | yes | no | yes | no | no |
| 4. (*n* = 3; *n* = 2/1) | no | yes | yes | no | no |
| 5. (*n* = 3; *n* = 2/1) | yes | no | no | no | no |
| 6. (*n* = 2; *n* = 1/1) | no | yes | yes | yes | no |
| 7. (*n* = 2; *n* = 1/1) | no | no | no | no | yes |
| 8. (*n* = 1; *n* = 1/0) | yes | no | no | no | yes |
| 9. (*n* = 1; *n* = 1/0) | no | yes | yes | yes | yes |
| Total number of cases: *n* = 30; first/second phase: *n* = 18/12 | | | | | |

*Note.* [a] For each strata, (the biggest) half of the cases, consisting of the most rich cases, were included in the first phase of analysis and the remaining cases were included in the second phase of analysis.

Table S3

*Overview of quotes, illustrating the results on the functionality of young women's behaviour prior to and four years after youth detention*

| | Before detention | | After detention |
|---|---|---|---|
| | ***Importance and content*** | | |
| Quote 1 | Independence as the most important basic human need<br>*Yeah, that was … that was actually what it was all about. I just wanted to do whatever I wanted to do and no one would stop me: not the police, not the judge, not the Youth Detention Centre, no one. (…) All the things that I was allowed to do, I didn't. And all the things that I wasn't allowed to do, I did. That was really how it was.* (Alex, 22 years old) | Quote 17 | Relatedness as the most important basic human need<br>*That (my daughter) is the breath of my life. If I were to lose her, I can assure you, I wouldn't do well.* (Julia, 19 years old) |
| Quote 2 | Relatedness as the most important basic human needs<br>*I was mainly looking for love, I think. Maternal love, that was what I was looking for. And that was the thing I was missing, very badly. (...) And that was also the thing I used to rack my brain about in the past, and that would keep me up at night.* (Chelsea, 22 years old) | Quote 18 | Independence and inner peace as the most important basic human needs<br>*That was very important to me, to have my own spot where I can unwind. Where I can come and go as I like, because it's mine. (…) So, yeah, that's what is most important to me: to come and go, how and where and when I want.* (Ruth, 19 years old) |
| | ***Internal/external resources/obstacles (Flaw 1)*** | | |
| Quote 3 | Particular skills as internal resources for fulfilling the need of independence<br>*I remember that once others were saying to me "come on, we are going to plunder the* | Quote 19 | Daily living skills and persistency as internal resources for fulfilling the need of independence<br>*I always carried my own weight. That's how it is. Yeah, there aren't many people I can count* |

| | | | |
|---|---|---|---|
| | *counter of a night shop, or something like that". But then I thought "okay, let us stop, enough is enough".* (Lynn, 19 years old) | | *on. And that's how you learn to carry your own weight. (...) It works out, yet with difficulty. But tough is also possible. At least, that's what I think.* (Stacy, 22 years old) |
| Quote 4 | Specific leisure interests as internal resources for fulfilling the need of excellence at play<br>*Yeah, back then, I was drawing already. Cause even in the Youth Detention Center I had, my room was stuffed with the drawings I had been drawing back then. Yeah, I really often engaged myself, in music and dance and so on.* (Ariana, 20 years old) | Quote 20 | Emotion regulation skills as internal resources for fulfilling the need of inner peace<br>*(...) Now, I am starting to be able to talk about things in the past. Back then, I wouldn't say what had happened. (...) I still remember very well. I would never tell anything in great detail. Now, I am starting to ... My life, it is my life, I lived it, I also should be able to talk about it.* (Melissa, 21 years old) |
| Quote 5 | Negative cognitions/affects as internal obstacles to fulfilling the need of inner peace<br>*'Psychological health' was also not good, cause my psychological health was nothing else but depression, negativity inside my head.* (Melissa, 21 years old) | Quote 21 | Physical injuries as internal obstacles for fulfilling the need of physical health<br>*I have always had this problem with my back. (...) I am still suffering from that, now and then. (...) Thanks to my ex-boyfriend, he pushed me. (...). So, yeah, I landed badly, and, yeah, I didn't go to the hospital in time (...). (*Melissa, 21 years old) |
| Quote 6 | Behavioural issues as internal obstacles to the fulfilment of excellence in school/work<br>*Back then, I was running away and doing things other than school. While actually I wanted to go to school, but, yeah, when you run away and you are going to school, you get caught. So, yeah, that was no option.* (Ruth, 19 years old) | Quote 22 | Anxiety as an internal obstacle for fulfilling the need of inner peace<br>*When I am too far away from home, I get, how should I say this, I get panic attacks, because I... (...) Before the YDC I also resided in psychiatry [inpatient psychiatric care]. So, I have spent so many years inside that, now, when I am too far away from home, I can't keep it together, I get stuck.* (Monica, 20 years old) |
| Quote 7 | Disconnection from family as external obstacle to fulfilling the need of relatedness | Quote 23 | Presence/support from one's partner/family in law as an external resource for fulfilling the need of inner peace |

| | | | |
|---|---|---|---|
| | *I was also, because I missed the security and affection, I was also really desperately looking for love, for someone who cherished me, who made me feel good, who was there for me.* (Sophie, 19 years old) | | *Now, I don't have my family members who humiliate me. My boyfriend loves me as I am. His family also tells me "hey, you are who you are, we love you as you are". (...) Before, I was thinking "okay, I will have to do something about this, because of this and that". But, now, I don't have this feeling anymore, cause those people are no longer part of my life. I am more, more at ease now.* (Monica, 20 years old) |
| Quote 8 | Placement in institutions with strict rules as external obstacle for fulfilling the need of independence<br>*But, because of the difficulties at home back then, yeah, it started: facility, facility, ... And I wasn't used to that and I didn't like it that all of a sudden I had to go to a facility. And I even didn't know the reason. Even now, I still don't know. No, really, I didn't know the reason myself. Even now, I still don't. And, I think that's just terrible, cause I do not choose to go to a facility. If I were to choose, I would simply be at home.* (Lizzie, 18 years old) | | |
| **(In)appropriate means/goals and their relationship to antisocial behaviour (Flaw 2)** | | | |
| Quote 9 | Seeking social support as an appropriate means to fulfil the need of inner peace<br>*I wanted to leave home. I didn't run away. But, at that moment, yeah, my father had beaten me and so on. And so I had to go and I went all the way by foot to my grandmother. I walked about eleven kilometers to my grandmother. Really, really terrible. And I filed a complaint against my father, cause he had really severely abused me.* (Hannah, 18 years old) | Quote 24 | Listening to music as an appropriate mean to fulfil the need of inner peace<br>*Cause music is also a way of processing things. (...) Yeah, I am listening to music about two hours a day. (...) And, I don't know, apparently it helps me to feel better. Or, when I am too energetic, (it helps me) to cool down. It always helps me to keep my emotions balanced.* (Alex, 22 years old) |

| | | | |
|---|---|---|---|
| Quote 10 | Engagement in healthy/supportive relationships with others as an appropriate mean to fulfil the need of relatedness<br>*I didn't grow up at home. I grew up in facilities, so I don't know what an upbringing at home can be like. I don't know. To me, these facilities were a first home, cause I resided there from the age of two and a half years old until the age of fifteen. And to me, they [the caregivers in the facility] were my mamas and papas.* (Alice, 22 years old) | Quote 25 | Engagement in healthy/supportive relationships with one's partner/family as an appropriate mean to fulfil the need of relatedness<br>*We (my partner and I) understand each other, we are able to complement each other well, we are able to support each other in all the things we do, we respect each other, we trust each other. And, yeah, that's how it has been for two years now. And, to me, it can continue like that for still a very long period of time. (...) And my sister, she's not only your sister, but also your second mother. (...) Cause, in fact, she had my back. In fact, she took care of me.* (Alice, 22 years old) |
| Quote 11 | Substance use as an inappropriate mean to fulfil the need of inner peace<br>*Arguments, being beaten all day. Yeah, I really tried to escape. I tried everything to escape. I started cutting myself to escape, to be able to concentrate my pain a little bit faster (...). I smoked joints, I smoked joints until I couldn't stand on my own feet, until I was so stoned that I was laughing about anything, just so I wouldn't have to think about it anymore.* (Melissa, 21 years old) | Quote 26 | Substance use as an inappropriate mean to fulfil the need of inner peace<br>*You forget everything. Look, sometimes, sometimes… Each night I smoke three or four (joints) one after the other. (...) Then I put my duvet over here and I sleep like a log.* (Meg, 19 years old) |
| Quote 12 | Spiral effect instigated by running away<br>*I was running away from my own problems and I was actually always looking for something better. Which, actually, always was something worse. Cause I actually also ended up with guys and so on (...) Sometimes, when you are searched by the police, you have to find a place* | Quote 27 | Use of aggression as an inappropriate mean to fulfil the need of independence<br>*If I say "no, get off of me", then I mean "get off of me". End of story. "Just let me go". That's how it works. When I say "no", then it's "no". If I want to walk out this door, I will walk out this door. If someone is standing in front of it, I will kick down this door, I will really kick it down. And that's "pook, toonk, pook" and it's over.* (Joanna, 22 years old) |

| | | | |
|---|---|---|---|
| | *to sleep. In that case, where do you end up? With junkies. Really, most people you are staying with are junkies. So, you end up seeing things that aren't really good.* (Alex, 22 years old) | | |
| Quote 13 | Spiral effect instigated by engaging in unhealthy romantic relationships<br>*He (my boyfriend) was the reason why I simply started going down a bad path. Before, I was actually okay. I did fine, I went to school. Actually, I did everything well. (...) But that's actually the thing that started everything. And in the beginning, it looked like a healthy relationship, which really in the end only got worse.* (Rebecca, 21 years old) | | |
| | ***(Lack of) scope (Flaw 3)*** | | |
| Quote 14 | Lack of scope<br>*I don't care. I wasn't thinking. Not at any point. Yeah, I also didn't have a lot of people left anymore. Then, I just had my boyfriend, yeah. But he had a very bad influence on me. And, at that moment, he seemed to be my everything. I would have done everything for him, really everything. (...) I guess I indeed must have really clung to him, that I really couldn't see anything anymore.* (Rebecca, 21 years old) | | |
| | ***Coherence/conflict (Flaw 4)*** | | |
| Quote 15 | Conflict between the needs of independence and relatedness<br>*Back then, it was a little bit harder, I guess, to make my own choices and so on. Yeah, cause sometimes I didn't want to run away. But sometimes, yeah, he (my boyfriend) was nagging and nagging, so that I gave way in the end.* (Stacy, 22 years old) | Quote 28 | Coherence between the needs of inner peace and relatedness<br>*Lately, she (my mother) asked my sister once "why isn't she calling me?". I answered very clearly "because I don't know whether she is a good thing in my life or a bad thing in my* |

| | | | |
|---|---|---|---|
| | | | *life". But eventually I have in fact decided. I simply think "the more she's out of my life, the better". She would only bring out the worst in me.* (Rebecca, 21 years old) |
| Quote 16 | Conflict between the needs of independence and belonging<br>*But, looking back, actually I didn't yet make my own choices back then, cause my friends made my choices, they made the choices for me. Cause if they said "we are going to the park", yeah, then we eventually went to the park. Then I didn't say "yeah, no, I am going there [elsewhere]". That was, yeah, then you are following your group. And, yeah, then you are not making the choices yourself that you actually probably would want to make.* (Lynn, 19 years old) | Quote 29 | Coherence between the needs of independence and relatedness<br>*To me, that's also very important in my relationship. It's not that I am together with my boyfriend because I need him. I do not need him, I am together with him because I want to be together with him. And that's very important to me. (...) I have my own apartment (...) If we have an argument or something like that, "do your thing", but I have, I have my own life. (...) I am also happy about that, that makes a relationship healthy, I think, I am 100 percent sure of it. That you're not, that nobody is dependent upon anybody.* (Ruth, 19 years old) |
| | | | ***Turning points*** |
| | | Quote 30 | Increased self-care as a turning point<br>*I think the powerful thing is that I do keep my head above water to some extent. In the past, I always backed up others and I always drowned myself. And now I'm like "first, think about yourself and, afterwards, help the other". Cause, of course, when you're not doing well yourself, you can't help anybody else.* (Lisa, 18 years old) |
| | | Quote 31 | Support from one's partner as a turning point<br>*I had just left the YDC [Youth Detention Centre], back then, I was still living my, my street life. And he (my boyfriend) actually, he actually helped me, supported me, so I ... so I became* |

| | |
|---|---|
| | *the woman that I actually… so I became a real woman. Do you understand? Not hanging out on the street all the time, sitting outside, drinking, partying, hanging around with bad people. Yeah, he really pulled me out of it. At my pace, not at his, at mine. (…) That was really what I needed, that someone whom I loved told me "(the young women's first name), you're not on the right path. You have to stop or else it will never get better".* (Ruth, 19 years old) |
| Quote 32 | The choice to live a clean life as a turning point<br>*It is simply your own switch you have to make inside your head. Suddenly, you have to say "so be it, I'm leaving it (the drugs) behind, I can do this, I have goals I want to leave it behind for". And if you have those goals, such as a child, a good relationship, a proper house of your own, then, all of a sudden, it was possible for me to do so.* (Chelsea, 22 years old) |
