# Supplementary material for: A Qualitative Study on Young Women’s Lives Prior to and Four Years after Youth Detention: Examining the Good Lives Model’s Aetiological Assumptions
Source: Int J Environ Res Public Health. 2021 Nov 11;18(22):11830. doi: 10.3390/ijerph182211830 (PMC8625703; doi:10.3390/ijerph182211830)
Supplement: Supplementary file 1 [file ijerph-18-11830-s001.zip › 1. Supplementary text.pdf]

International Journal of
Environmental Research and Public Health

MDPI

Supplementary text

Text S1: Recruited participants of the baseline sample (T0)

Between February 2012 and June 2014, a total of 215 female adolescents were admitted to the YDC. DFA could participate if the following criteria were met: (i) adjudicated to be detained for at least one month; (ii) sufficient knowledge of Dutch; and (iii) sufficient cognitive abilities. The latter criteria were based upon the assessment of both staff and interviewer of the adolescent's ability to participate in Dutch conversations and to read and understand the informed consent form. Based on these criteria, 46 of them were excluded: 11 were adjudicated to be detained for less than one month, 28 did not have sufficient knowledge of Dutch, and seven did not have sufficient cognitive abilities. In total, 169 DFA were allowed to participate. Two of them could not be approached due to acute psychiatric crisis, and 20 of them and/or their parents declined participation, which resulted in a baseline sample of 147 participants (participation rate = 86.98%). Study participants ($N = 147$) did not display significant differences from those who(se parents) declined participation in the study ($N = 20$) with regard to age, ethnic origin, and detention history (details available upon request from the first author). At T0, participants were between 13.51 and 17.91 years old ($M = 16.22$; $SD = 1.09$), were predominantly of Belgian origin (65.31%), and 20.41% had been detained in the past.

Text S2: Longitudinal research design (T0-T4)

Baseline assessment took place within the first three weeks after admission (T0; $N = 147$; quantitative). Follow-up assessments took place one and two months after baseline (T1 and T2; $n = 124$ and $n = 108$; quantitative); six months after discharge (T3; $n = 103$; quantitative); and four years after discharge (T4; $n = 50$ of which $n = 49$ quantitative and $n = 30$ qualitative). The quantitative T0-T3 data have been used in prior work (Blinded for review). The present study is one of the first to report on the qualitative T4 data.

Text S3: Differences between T4 (*n* = 30) and remaining (*n* = 117) participants

Differences between T4 participants (*n* = 30) and the remaining (*n* = 117) participants are presented in Table S1. T4 participants (*n* = 30) did not display significant differences from the remaining participants (*n* = 117) with regard to age, socioeconomic status, intact family, school attendance, past detention history, any psychiatric disorder/ any externalizing disorder/ pure internalizing disorders, and self-reported offending at baseline. Yet, T4 participants, compared to the remaining participants, did show a significantly higher percentage of adolescents of Belgian origin, a higher percentage of adolescents with any internalizing disorder/ any comorbidity/ comorbidity of internalizing and externalizing disorders, but a lower percentage of adolescents with pure externalizing disorders at baseline. In addition, T4 participants displayed significantly lower QoL scores at baseline for all domains (i.e., physical health, psychological health, social relationships, and environment) compared to the remaining participants.

Text S4: Recruitment procedure of the baseline sample (T0)

At T0, DFA who were eligible to participate were approached and received oral and written information about the aims, content, and duration of the study. They were guaranteed that their information would be treated confidentially and that refusal to participate would not affect their judicial status or stay in the YDC. Before starting the assessment, written informed consent was given by the participants. A letter with information about the aims and practical aspects of the study was sent to DFA's parents and they could decline participation (i.e., passive informed consent). At the end of T0, 124 participants provided written informed consent to contact them for further research. They also provided detailed contact information (e.g., of themselves, family, friends, their court consultant) in order to facilitate tracing them.
